# Andexanet Alfa for Reversal of Factor Xa Inhibitors in Intracranial Hemorrhage: Observational Cohort Study

**DOI:** 10.3390/jcm11123399

**Published:** 2022-06-13

**Authors:** Sebastian Rauch, Hans-Peter Müller, Jens Dreyhaupt, Albert C. Ludolph, Jan Kassubek, Katharina Althaus

**Affiliations:** 1 Department of Neurology, University of Ulm, 89081 Ulm, Germany; sebastian.rauch@uni-ulm.de (S.R.); hans-peter.mueller@uni-ulm.de (H.-P.M.); albert.ludolph@rku.de (A.C.L.); jan.kassubek@uni-ulm.de (J.K.); 2 Institute of Epidemiology and Medical Biometry, University of Ulm, 89081 Ulm, Germany; jens.dreyhaupt@uni-ulm.de

**Keywords:** coagulants, factor Xa inhibitors, hemorrhage, intracranial hemorrhages, antidote, andexanet alfa, antithrombotic drugs, hemostasis, thrombosis, magnetic resonance imaging

## Abstract

Background: Intracranial hemorrhage (ICH) is associated with high mortality and morbidity, especially in patients under anticoagulative treatment. Andexanet alfa (AA) is a modified recombinant form of human factor Xa (FXa) developed for reversal of FXa-inhibitors, e.g., in the event of ICH, but experience is still limited. Methods: This monocentric retrospective observational cohort study included 46 patients with acute FXa-inhibitor-associated non-traumatic ICH (FXa-I-ICH) of whom 23 were treated with AA within 12 h after symptom onset, compared to 23 patients with usual care (UC). Volumetrically analyzed hematoma expansion (HE) in brain imaging, clinical outcome and incidence of adverse events were analyzed. Results: All patients (mean age 79.8 ± 7.2 years) were effectively anticoagulated. The cohort included severely ill patients with large hematoma volumes (median 20.4, IQR 7.8–39.0 mL). Efficacy, as assessed by HE in imaging, was very good in the AA-group. There was no (0.0%) relevant HE (>33%) in contrast to UC-group (26.1%). Nevertheless, we observed a high incidence of thromboembolic events (30.4% vs. 4.4%) and non-favorable outcomes (death/palliative condition) in 43.5% vs. 26.1%. Conclusions: There was no HE in the volumetric neuroimaging assessment in the AA-group, but clinical outcomes remained often worse. Large randomized trials for the use of AA in patients with acute FXa-inhibitor-associated ICH are needed to investigate the clinical outcome in consideration of the rates of thromboembolism.

## 1. Introduction

Although non-vitamin-K-antagonist oral anticoagulants (NOACs) have lower rates of bleeding complications in comparison to warfarin, intracerebral hemorrhage (ICH) is still associated with high mortality and morbidity, especially in these patients under anticoagulative treatment [1,2]. In patients with NOAC-associated ICH current American and international guidelines do not provide strong recommendations for specific reversal strategies [2,3]. However, there is converging evidence in Vitamin-K-Antagonists (VKA)-associated ICH that an acute reversal to international normalized ratio-(INR) levels <1.3, due to favor 4-factor prothrombin complex concentrates (PCC), positively influences clinical outcomes [4,5]. In patients with ICH under anticoagulation with dabigatran, reversal of anticoagulation effects can be achieved within minutes by application of idarucizumab, a humanized Fab fragment of a monoclonal antibody which specifically binds dabigatran with very high affinity [6] and seems to prevent HE in patients with a dabigatran-associated ICH without severe side effects [7,8]. For patients with factor Xa (FXa) inhibitor-associated bleedings, andexanet alfa (AA), a modified recombinant inactive form of human FXa, was developed for reversal [9]. The dosage and the application speed of the AA infusion was patient-specific, depending on which FXa inhibitor was taken, the prescribed dosage of the FXa inhibitor, and the latency between its last intake and the start of the AA treatment. AA acts as a human decoy receptor binding to the active site of FXa inhibitors with high affinity and possesses no catalytic activity [9,10]. The ANNEXA-4 study evaluated 352 patients presenting with acute major bleeding treated with AA and showed a median FXa activity reduction of 92% [9]. AA was approved by the FDA in 2018 and the EMA in 2019 for rivaroxaban and apixaban reversal [10,11]. The approvals were linked to the implementation of a study to determine the efficacy and safety of AA compared to standard care so that, in June 2019, the ANNEXA-I study began (NCT03661528).

Patients with ICH continue to bleed after the initial hemorrhage, known as hematoma expansion (HE) which is a well-established independent predictor of clinical deterioration [12]. HE represents a plausible therapeutic target in many clinical trials due to the characteristic of being potentially preventable and as such, is a very important outcome predictor [12]. HE is defined as a more than 33% increase in ICH volume within the first 24 h resulting in significant neurological deficits and enhancement of ICH-induced primary and secondary brain injury [13]. Active blood pressure reduction has proven to be an effective therapy for ICH due to a risk reduction of HE and rebleeding [14,15]. However, HE can occur for more than 24 hours in anticoagulated patients, if anticoagulation status is not reversed [16,17].

In light of this clinical constellation, AA seems to have a sufficient effect on hemostasis, but several questions remain unanswered: the role of the hemostatic rebound, the HE rates and the relation of the possible benefit to the risk of prothrombotic complications. This single-center observational cohort study reports the experience with AA in patients with FXa-inhibitors-associated ICH (FXa-I-ICH) with regard to efficacy and safety.

## 2. Materials and Methods

### 2.1. Patients

We conducted a retrospective, single-center observational study that included a consecutive series of patients with an acute FXa-inhibitors-associated non-traumatic ICH treated with Andexanet alfa or usual care within 12 h after symptom onset who were admitted to the Department of Neurology of the University of Ulm, Germany between 01/2016 and 12/2021. Palliative patients were excluded from analyses. By systematic patient chart review, we collected baseline characteristics including cardiovascular risk factors, laboratory and coagulation parameters inclusive anti-Xa activity (commercial assays as part of standardized laboratory diagnostics to measure the concentration of rivaroxaban, apixaban and edoxaban, respectively in citrated patient plasma; BIOPHEN™ DiXaI kit, HYPHEN BioMed, Neuville-Sur-Oise, France) together with magnetic resonance imaging (MRI) or computed tomography (CT) scans from all patients. From all patients treated with AA, cerebral imaging after reversal and side effects including thromboembolic and ischemic events until discharge from hospital were collected. Clinical outcome was assessed using the National Institutes of Health Stroke Scale (NIH Stroke Scale, NIHSS) [18] and modified Rankin scale (mRS) [19] at discharge.

We have differentiated between efficacy and safety endpoints, respectively. Primary efficacy endpoint was hematoma expansion >33% in andexanet alfa treated patients versus usual care (UC) [9,13,20], while secondary efficacy endpoint was neurological function at discharge.

Primary safety endpoint was the occurrence of thrombotic events, and the secondary safety endpoint was in-hospital mortality or palliative course. 

AA was dosed according to the product labeling for life-threatening bleeding associated with FXa-inhibitors with a bolus, followed by a 2-h infusion [21]. All patients were treated in our stroke unit according to the national guidelines. For acute blood pressure management, we aimed at a systolic blood pressure <140 mmHg.

The retrospective data analysis was approved by the Ethics Committee of the University of Ulm, Germany (reference 23/21).

### 2.2. Imaging

**Figure 1:**
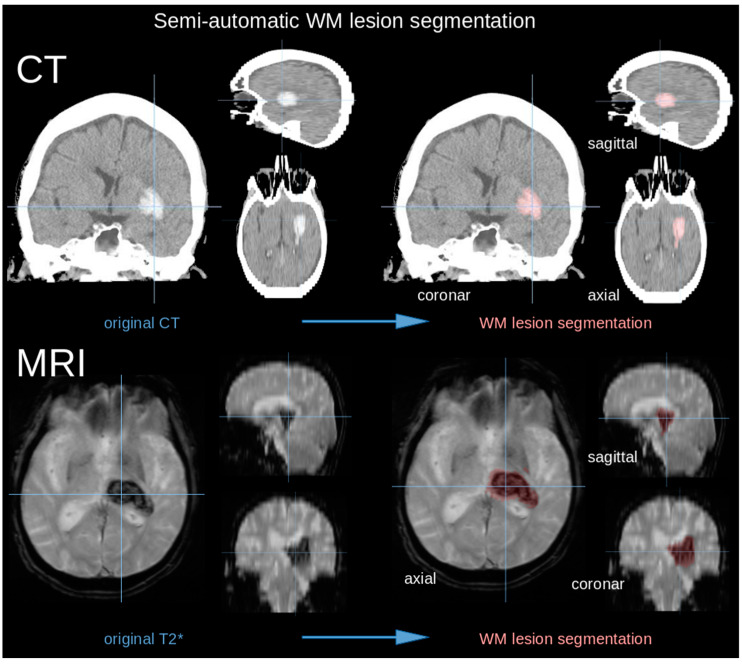
(**Upper panel**) CT scan in coronar, axial, and sagittal slice representation (see crosshairs) with colored infarct area (right). (**Lower panel**) MRI scan in axial, sagittal, and coronal slice representation (see crosshairs) with segmented infarct area (right).

The database consisted of standard clinical magnetic resonance imaging (MRI) protocols acquired on a 1.5 Tesla MRI (Magnetom TIM Symphony, Siemens, Erlangen, Germany) or, in case of contraindication for MRI, computed tomography (CT) (Somatom Emotion 16 eco, Siemens, Erlangen, Germany) [22]. The T2* MRI scans consisted of 40–50 coronar slices (thickness 3.0 mm) with an in-plane resolution of 0.43 × 0.43 mm^2^; the CT protocol consisted of 40 axial slices (thickness 5.0 mm) with an in-plane resolution of 0.33 × 0.33 mm^2^. Hematoma volume was measured on MRI or CT scans. The Tensor Imaging and Fiber Tracking (TIFT) software was used for processing of the imaging data [23,24,25]. MRI or CT scans were transformed into a 3-dimensional isotropic grid with a size resolution of 0.5 mm. For the volumetric measurement of ICH, lesion-related voxels were identified by an operator-defined intensity range using semiautomatic delineation of the hyperdense bleeding tissue in CT or hypointensity in T2*-MRI (Figure 1). Patients with surgical hematoma evacuation were excluded from the measurement of the follow-up images due to the lack of comparability.

### 2.3. Statistical Analysis

Continuous data were reported as mean, standard deviation or median and quartiles as appropriate and min-max. Ordinal and categorical data were analyzed as absolute and relative frequencies. For group comparisons of continuous data, the two-sample *t*-test or the Wilcoxon rank sum test was used. Group comparisons of categorical data were done with the chi square test or Fishers exact test as appropriate. A two-sided *p* value ≤ 0.05 was considered to be statistically significant. Because of the explorative nature of this study, all results from statistical tests have to be interpreted as hypothesis-generating and not as confirmatory. An adjustment for multiple testing was not made. Statistical analysis was performed with SAS, version 9.4 (SAS Institute, Cary, NC, USA).

## 3. Results

### 3.1. Baseline Demographics

**Figure 2:**
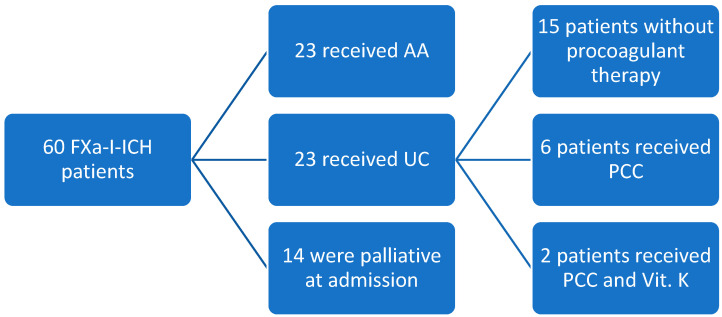
Flow chart of all FXa-I-ICH patients. FXa-I-ICH indicates patients with acute ICH on factor-Xa-inhibitors. ICH: intracerebral hemorrhage; AA: andexanet alfa; UC: usual care; PCC: favor 4-factor prothombin complex concentrates; Vit K: Vitamin K.

During the investigation period, we screened 349 patients with an ICH. A total of 60 patients out of this sample had an FXa-inhibitors-associated ICH (FXa-I-ICH). Fourteen patients only received palliative care at admission and were excluded from further analyses. Of the remaining 46 patients (mean age of 79.8 ± 7.2 years), 23 were treated with andexanet alfa, 23 were treated with usual care (Figure 2).

Most of the 46 patients had substantial cardiovascular disease; details are summarized in Table 1. Median NIHSS was 10.0 (IQR 4.0–20.0), the AA patients (NIHSS 11.0, IQR 9.0–21.0) were more severely affected (*p* = 0.03) compared to the UC patients (NIHSS 7.0 IQR 2.0–19.0). Most of the FXa-I-ICH patients were on rivaroxaban (*n* = 27, 58.7%), 14 (30.4%) on apixaban, and 5 (10.9%) on edoxaban. Median anti-Xa activity at admission was 147.0 ng/mL (IQR 58.7–241.0) for rivaroxaban, 96.4 ng/mL (IQR 66.3–138.3) for apixaban, and 26.2 ng/mL for edoxaban (only 3 measurements available: 16.0, 26.2, 372.0 ng/mL, respectively. Indication of FXa-inhibition intake over all FXa-I-ICH patients was atrial fibrillation in 87.0%, venous thromboembolic disease in 17.4% and in one case unavailable. A total of 23 (50.0%) patients were treated with AA, 23 (50.0%) patients with usual care: 6 (26.1%) of them received PCC, 2 (8.7%) received PCC and vitamin K and 15 patients (25.2%) were treated without procoagulant medication (Figure 2).

Median hematoma volume at baseline did not differ between the two groups (*p* = 0.46) with 19.8 mL (IQR 11.4–50.0) in the AA-group and 24.7 mL (IQR 6.5–36.1) in the UC-group. Most baseline characteristics were equally balanced between the AA- and UC-group. Details on baseline characteristics are summarized in Table 1.

### 3.2. Efficacy and Safety

Follow-up imaging was available in 91.3% (*n* = 21) of the AA group and 82.6% *(n* = 19) of the UC group. 6 patients were lost from follow-up imaging, because of palliative course or death. In all patients treated with AA with follow-up imaging available, median hematoma volume remained constant from baseline (19.8 mL) to the follow-up imaging (20.8 mL). No patient had an HE > 33%. In the UC-group, median HE became smaller, but 6 of the 19 patients (31.6%) with a follow-up imaging had a HE > 33%. The rate of deceased or palliative patients was not significantly increased in the AA group (*n* = 10; 43.5%) compared to the UC group (*n* = 6; 26.1%). Two patients had a good clinical outcome (mRS ≤ 3) at discharge from acute hospital (8.7%) in the AA group versus 9 patients (39.1%) in the UC group (*p* = 0.02). Median NIHSS at discharge was 13 (IQR 6.0–42.0) in the AA group and 4.0 (IQR 2.0–42.0) in the UC group (*p* = 0.05), respectively.

Acute thromboembolic events occurred in 7 (30.4%) of the AA patients, all in the absence of re-anticoagulation. These patients had ischemic infarcts with mostly embolic patterns in the follow-up MRI, 5 (71.4%) without new neurological symptoms. Three patients had an ischemic stroke and simultaneously a non-ST elevation myocardial infarction (NSTEMI). In the UC group, one patient had a re-stroke in absence of re-anticoagulation (4.3%). 34.8% (*n* = 8 one with COVID-19) of all AA patients and 17.4% (*n* = 4) of the UC patients (*p* = 0.18) developed pneumonia, which was not rated as causally related to AA. Outcome details are summarized in Table 2.

## 4. Discussion

The current retrospective, single-center, observational study included 46 patients with an acute FXa-inhibitor-associated, non-traumatic ICH and evaluated efficacy and safety of reversal the FXa inhibitors with andexanet alfa compared to usual care. In addition to the registration study and some other retrospective studies, there is little experience with AA till today [9,25,26,27].

All patients of our cohort had high serum levels of anti-FXa activity of the respective NOAC on admission. Our cohort includes differently affected patients with hematoma volumes on admission ranging from small to very large (min 2.3–max 132.5 mL) with a median baseline hematoma volume in the AA group of 19.8 mL (IQR 11.5–50.0 mL), and 24.7 mL (IQR 6.5–36.1 mL) in the UC group and thus constitutes real-world data. We also treated patients with known risk factors for hematoma expansion, such as larger ICH volume or high NIH Stroke Score at presentation, or low probability of survival at 30 days. In contrast, the approval Annexa-4-study included patients without relevant anti-FXa activity levels and excluded patients with a bleeding volume >60 mL. The median baseline volume in the Annexa-4-study was only 9.4 mL (3.3–20.8) [9], similar to other studies from Ammar et al. [26] or Barra et al. [27].

Since there are different measurement methods and definitions for the extent of the hematoma, [9,13,20,26] we opted for a standardized volumetric assessment [23]. According to the established criteria for HE [9,13,20], 100% of our patients in the AA group had no significant HE compared to 6 patients in the UC group.

The incidence of new thromboembolic events was significantly higher in the AA group in contrast to the UC group with significantly more ischemic strokes which were clinically silent in 5 patients. A possible thromboembolic effect must be discussed. Additionally, the number of thromboembolic events in our AA group were therefore also significantly higher than reported in the ANNEXA-4 study (30.4% vs. 10%). The very high proportion of MRI-based follow-up imaging might be regarded as a probable reason for the significantly higher number of clinically silent ischemic infarcts that would have been missed in a CT scan. However, it must also be discussed whether we had higher ischemic stroke rates due to the more severely affected patients, since no thromboembolic events occurred in the studies on AA with healthy subjects [10], but only occurred during examinations in the bleeding patient population [9,20].

The rate of other thromboembolic events is consistent with previous studies [9,20,26,27]. The clinical outcome (measured in NIHSS and mRS) of the patients in our cohort was worse and the mortality/palliative condition rate was substantially higher (whole group 34.8%, AA group 43.5% and UC group 26.1%) than in the ANNEXA-4 study (14%) [9]. This can easily be explained by the significantly more severely affected patients, some of whom had very large ICHs. When comparing the results of our single-center observational study to previously reported studies, it is important to take the variability in treatment patterns, the mechanism of ICH and baseline severity of clinical deficits into consideration. Likewise, the outcome in our AA group was worse than in the UC group. However, interpretation has to be done with caution, since these were not randomized groups and hematoma characteristics like the location of the bleeding was not included in the analysis.

Our observational study had several limitations. In addition to the general limitations of the retrospective study design we have a single-center data base with potential bias. Despite the inclusion of a control group, we can only identify possible trends between the patients treated with andexanet alfa compared to usual care due to the lack of randomization and different treatment regimes in the UC group. The ongoing randomized, controlled ANNEXA-I trial is evaluating the efficacy and safety of AA versus the usual standard of care/PCC in patients with ICH anticoagulated with FXa-inhibitors (NCT03661528) and will hopefully answer this important question. Another limitation is the lack of inclusion of the exact symptom-to-treatment times in the evaluation. However, only patients with an onset of symptoms less than 12 h before AA administration were included.

A strength of our study is that all treated patients showed relevant anti-FXa levels and thus, different to the ANNEXA-A study (in which 1/3 of the patients showed no relevant anti-FXa activity), only anticoagulated patients were treated.

## 5. Conclusions

The decision to treat patients in a clinical setting, i.e., beyond the standardized procedures of a clinical trial, led to the treatment of very severely affected patients for whom no treatment evidence from previous studies existed.

In patients with acute ICH under use of an FXa inhibitor, no significant hematoma expansion occurred after andexanet alfa administration compared to the usual care patients. Unfortunately, thromboembolism (ischemic stroke or myocardial infarction) occurred in 30% within four days after reversal. Large randomized studies comparing the efficacy and safety of AA in patients with acute FXa-inhibitor-associated ICH are needed to investigate whether preventing HE improves clinical outcome despite the high rates of thromboembolism.

## Figures and Tables

**Table 1:** Baseline Characteristics.

	Overall FXa-I-ICH * (n = 46)	FXa-I-ICH with AA Treatment (n = 23)	FXa-I-ICH with UC (n = 23)	*p*-Value
Age, y, mean ± SD	79.8 ± 7.2	78.1 ± 7.3	81.5 ± 6.9	0.11
Male, *n* (%)	25 (54.4%)	14 (60.9%)	11 (47.8%)	0.37
risk factors *n* (%)				
aHT	42 (91.3%)	21 (91.3%)	21 (91.3%)	1.00
atrial fibrillation	40 (87.0%)	19 (82.6%)	21 (91.3%)	0.67
renal Insufficiency	6 (13.0%)	4 (17.4%)	2 (8.7%)	0.67
coronary heart disease	16 (34.8%)	8 (34.8%)	8 (34.8%)	1.00
Indication of FXa inhibition, *n* (%)				
Atrial fibrillation	40 (87.0%)	19 (82.6%)	21 (91.3%)	0.67
Venous thromboembolic disease	8 (17.4%)	6 (26.1%)	2 (8.7%)	0.24
Unclear indication	1 (2.2%)	0 (0.0%)	1 (4.3%)	1.00
FXa inhibitor therapy, *n* (%)				
Rivaroxaban	27 (58.7%)	17 (73.9%)	10 (43.5%)	0.12
Apixaban	14 (30.4%)	5 (21.7%)	9 (39.1%)	
Edoxaban	5 (10.9%)	1 (4.3%)	4 (17.4%)	
Scores, median (IQR)				
NIHSS score admission	10.0 (4.0–20.0)	11.0 (9.0–21.0)	7.0 (2.0–19.0)	0.03
Modified Rankin Scale score ^‡^	2.0 (1.0–3.0)	3.0 (1.0–4.0)	1.0 (1.0–3.0)	0.06
Imaging at admission				
CT/MRI at admission *n* (%)	8 (17.4%)/38 (82.6%)	3 (13.0%)/20 (87.0%)	5 (21.7%)/18 (78.3%)	0.70
ICH volume, mL, Median (IQR) (min–max)	20.5 (9.4–37.1) (2.3–132.5)	19.8 (11.4–50.0) (4.4–123.6)	24.7 (6.5–36.1) (2.3–132.5)	0.46
laboratory, median (IQR)				
INR	1.17 (1.08–1.27)	1.11 (1.11–1.27)	1.17 (1.04–1.30)	0.48
PTT, s	33.7 (30.8–36.7)	33.4 (30.3–36.7)	33.8 (31.7–40.2)	0.69
Anti-FXa-activity				
Rivaroxaban (ng/mL)	147.0 (58.7–241.0)	166.7 (93.1–241.0)	102.9 (33.3–233.8)	0.38
Apixaban (ng/mL)	96.4 (66.3–138.3)	100.8 (85.8–121.8)	92.0 (66.3–196.6)	1.00
Edoxaban ^§^ (ng/mL)	16.0, 26.2, 372.0	-	16.0, 26.2, 372.0	-

Abbreviations: FXa-I-ICH: factor-Xa-inhibitors associated intracerebral hemorrhage; AA: andexanet alfa; UC: usual care; SD: standard deviation; IQR: interquartile range; min: minimum; max: maximum; NIHSS: National Institutes of Health Stroke Scale, CT: computer tomograph; MRI: Magnetic resonance imaging; INR: international normalized ratio; PTT: partial thromboplastin time; *n:* number of patients; y: years. * Overall FXa Inhibitors associated ICH excluded patients already palliative ad admission. ^‡^ Pre ICH. ^§^ the Anti-FXa-activity-level was only available for 3 out of 5 Edoxaban patients.

**Table 2:** Outcomes of FXa Inhibitors associated ICH treated with andexanet alfa.

	Overall FXa-I-ICH * (n = 46)	FXa-I-ICH with AA Treatment (n = 23)	FXa-I-ICH with UC Treatment (n = 23)	*p*-Value
**Imaging**				
CT/MRI follow-up *n* (%) ^‡^	16/24	6 (28.6%)/15 (71.4%)	10 (52.6%)/9 (47.4%)	0.12
Hematoma volume follow-up, ml; median (IQR) (min-max) ^‡^	20.0 (8.2–33.4) (1.7–139.8)	20.8 (11.2–35.2) (3.0–75.2)	14.5 (5.8–31.7) (1.7–139.8)	0.53
Number of patients with hematoma expansion >33%, *n* (%) ^‡^	6 (26.1%)	0 (0.0%)	6 (26.1%)	0.02
**Clinical outcome**				
Good outcome (mRS ≤ 3) on discharge *n* (%)	11 (23.9%)	2 (8.7%)	9 (39.1%)	0.02
NIHSS score on discharge median (IQR)	9.0 (2.0–42.0)	13.0 (6.0–42.0)	4.0 (2.0–42.0)	0.05
Death/palliative course on discharge *n* (%)	16 (34.8)	10 (43.5%)	6 (26.1%)	0.22
**Adverse events**				
Total thromboembolic events *n* (%)	8 (17.4%)	7 (30.4%)	1 (4.3%)	0.05
Ischemic stroke *n* (%)	8 (17.4%)	7 (30.4%)	1 (4.3%)	0.05
Myocardial infarction *n* (%)	3 (6.5%)	3 (13.0%)	0 (0.0%)	0.23
Both ^#^ *n* (%)	3 (6.5%)	3 (13.0%)	0 (0.0%)	0.23
Deep vein thrombosis *n* (%)	0 (0.0%)	0 (0.0%)	0 (0.0%)	
Pulmonary embolism *n* (%)	0 (0.0%)	0 (0.0%)	0 (0.0%)	
Pneumonia	12 (26.1%)	8 (34.8%)	4 (17.4%)	0.18

FXa-I-ICH: factor-Xa-inhibitors associated intracerebral hemorrhage, AA: andexanet alfa; UC: usual care; CT: computer tomograph; MRI: Magnetic resonance imaging; *n:* number of patients; IQR: interquartile range; min: minimum; max: maximum; NIHSS: National Institutes of Health Stroke Scale mRS: Modified Rankin Scale score. * Overall FXa Inhibitors associated ICH excluded patients already palliative ad admission. ^‡^ follow-up imaging is missing in 2 patients of the AA-group and 4 patients in UC-group. ^#^ Simultaneously within a person.

## Data Availability

Anonymized data will be shared by request from a qualified investigator.
